# Conflict of Interest in Clinical Practice Guideline Development: A Systematic Review

**DOI:** 10.1371/journal.pone.0025153

**Published:** 2011-10-19

**Authors:** Susan L. Norris, Haley K. Holmer, Lauren A. Ogden, Brittany U. Burda

**Affiliations:** 1 Department of Medical Informatics and Clinical Epidemiology, Oregon Health & Science University, Portland, Oregon, United States of America; 2 Kaiser Permanente Center for Health Research, Portland, Oregon, United States of America; University of British Columbia, Canada

## Abstract

**Background:**

There is an emerging literature on the existence and effect of industry relationships on physician and researcher behavior. Much less is known, however, about the effects of these relationships and other conflicts of interest (COI) on clinical practice guideline (CPG) development and recommendations. We performed a systematic review of the prevalence of COI and its effect on CPG recommendations.

**Methodology/Principal Findings:**

We searched Medline (1980 to March, 2011) for studies that examined the effect of COI on CPG development and/or recommendations. Data synthesis was qualitative. Twelve studies fulfilled inclusion criteria; 9 were conducted in the US. All studies reported on financial relationships of CPG authors with the pharmaceutical industry; 1 study also examined relationships with diagnostic testing and insurance companies. The majority of guidelines had authors with industry affiliations, including consultancies (authors with relationship, range 6–80%); research support (4–78%); equity/stock ownership (2–17%); or any COI (56–87%). Four studies reported multiple types of financial interactions for individual authors (number of types per author: range 2 to 10 or more). Data on the effect of COI on CPG recommendations were confined to case studies wherein authors with specific financial ties appeared to benefit from the related CPG recommendations. In a single study, few authors believed that their relationships influenced their recommendations. No studies reported on intellectual COI in CPGs.

**Conclusions/Significance:**

There are limited data describing the high prevalence of COI among CPG authors, and only case studies of the effect of COI on CPG recommendations. Further research is needed to explore this potential source of bias.

## Introduction

Since the emergence of the concept of evidence-based medicine [1], healthcare providers have sought ways to synthesize evidence into formats and products that are both valid and readily implemented into routine practice. According to the Institute of Medicine (IOM) 2008 report entitled *Knowing What Works in Healthcare* [2]: “Decisions about the care of individual patients should be based on the conscientious, explicit, and judicious use of the current best evidence on the effectiveness of clinical services.” Clinical practice guidelines (CPGs) are an important tool for achieving optimal patient care, and the recently updated definition of CPGs is “statements that include recommendations intended to optimize patient care that are informed by a systematic review of evidence and an assessment of the benefits and harms of alternative care options” [3]. Practice guidelines are increasingly common and can influence a large number of healthcare providers and patients [4], so their quality is critically important. There are data to suggest that CPGs improve processes of care [4]–[6] although data on the effectiveness of CPGs on health outcome are sparse and conflicting [6], [7].

Conflict of interest (COI) is one important potential source of bias in the development of CPGs. A COI is a set of conditions in which professional judgment concerning a primary interest (such as the health and well being of a patient or the validity of research), is unduly influenced by a secondary interest [8]. The secondary interests may be financial or nonfinancial. Bias almost always results in an overestimation of benefit and an underestimation of harm [9], and therefore biased CPGs can have profound implications for health care and ultimately patient outcomes. There is an emerging literature on the extent of COI, specifically industry relationships, in clinical research [10]–[17], and emerging empirical data suggest that financial relationships of the author or sponsor with industry are associated with study outcomes [10]–[12], [17] or decisions [16] favorable to the industry.

Although financial interests are often the most obvious, intellectual interests are increasingly recognized and may be powerful motivators for researchers, systematic reviewers, and guideline authors. Intellectual COI has been defined as “academic activities that create the potential for an attachment to a specific point of view that could unduly affect an individual's judgment about a specific recommendation” [18]. Intellectual interests include the advancement of medical science, as well as benefits from publication and the acquisition of research funding. Such interests are appropriate in themselves, but may conflict with the interests of research subjects and patients [19]. Levinsky compared financial to nonfinancial COI and described the latter as “more subtle yet more pervasive and [they] cannot be eliminated [19].” According to the American College of Cardiology Foundation and the American Heart Association [20] nonfinancial benefits from participation in clinical research trials include career advancement, fulfillment of a desire to do good, opportunity to publish, notoriety, invitations to present at meetings, future success in obtaining grant funding for research, research prizes, professional accolades for positive outcomes, and increased sense of self worth.

The objectives of this systematic review were to describe the extent of COI, both financial and intellectual, in CPGs and to examine the effect of COI on recommendations within CPGs. Outlining what is known as well as the gaps in evidence will help physicians and clinical researchers to: 1) critically appraise CPGs; 2) demand guidelines from their professional societies and other organizations that make every effort to disclose COI and minimize bias; 3) be cognizant of what is unknown with respect to this potential source of bias; and 4) to seek to reduce gaps in knowledge with future research.

## Methods

### Searching

We searched Medline (1980 to Week 4 March, 2011) for studies that examined the prevalence and effect of COI on the development and/or conclusions of CPGs, using the definition of CPG published by the Institute of Medicine [3]. Search terms included conflict of interest, drug industry, research support, and guidelines (clinical and practice), and Medical Subject Headings (MeSH) were combined with text words (see Table S1 for the search strategy). We also reviewed reference lists of included studies for additional citations. We did not restrict our search by language of publication.

### Selection

Studies were included if they examined either: 1) the prevalence of conflict of interest, industry relationships, funding, or sponsorship in CPGs or among guideline panel members and authors, or 2) the effect of such conflicts on guideline recommendations. CPGs could either focus on treatment or prevention interventions, or the use of diagnostic tests. Two authors (SLN and HH) independently identified potential studies from the literature search and consensus on inclusion was achieved. If consensus could not be reached between these two reviewers, a third coauthor was consulted (BUB) and consensus was achieved.

### Data Abstraction

One reviewer abstracted data into a predefined template that included domains for the CPG sponsor and clinical focus, study design, methods for data collection, prevalence and type of COI for CPGs and CPG authors, and data on the association between COI disclosures and recommendations. The resulting evidence tables were reviewed for accuracy by a second author. We did not perform quality assessment of the included studies because, although quality assessment tools are available for observational studies [21], we felt that formal assessment would not contribute to our ability to discriminate among studies in view of their descriptive, noncomparative designs.

### Data Synthesis

We undertook a qualitative synthesis across included studies because there was substantial heterogeneity with respect to characteristics and outcomes, making a meta-analysis inappropriate.

## Results

Twelve studies fulfilled inclusion criteria (Table 1) after 208 full-text publications were reviewed (Supplemental Information Figure S1). Nine studies were conducted in the US [13]–[15], [22]–[27], one in Greece [28], one in Australia [29], and one was international [30]. All studies examined COI in relatively recent guidelines, although one study searched for guidelines dating back to 1979 [28]. Several studies examined guidelines across a variety of specialties [14], [15], [22], [28], [29], while others focused on one or more guidelines within a medical specialty [13], [23]–[27], [30]. All included studies examined different cohorts of CPGs; no studies examined overlapping CPGs. Most studies focused on treatment or prevention CPGs, while two focused exclusively on diagnosis [26], [27].

**Table 1:** Characteristics of included studies.

AuthorYearCountry	CPG SponsorClinical Focus of Guidelines	No. CPGsNo. Authors	Study Design	Methods
**Guidelines on treatment or prevention**				
Buchan2010 [29]Australia	National Institute of Clinical StudiesVarious clinical conditions	313NR	Retrospective single-group cohort	Systematic search for Australian CPGs produced or reviewed between 2003 and 2007
Campbell2007 [14]US	NRNR	NR711	Cross-sectional	Survey of representative sample of US physicians
Choudhry2002 [15]North America and Europe	CPGs endorsed by North American and European societies on common adult diseases published 1991–1999Various adult diseases	44NR	Retrospective single-group cohort study and cross-sectional survey	Systematic review of Medline to identify CPGs; survey of CPG authors regarding specific financial interests
Cosgrove2009 [24]US	American Psychiatric AssociationSchizophrenia, 2004Bipolar disorder, 2002Major depressive disorder, 2000, 2005	320	Retrospective single-group cohort	Review of data from US Patent and Trademark Office, Lexis-Nexis Academic, Medline, other internet search engines, screening between 1989 and 2004 (DSM-IV was published in 1994)
Coyne2007 [23]US	Kidney and Dialysis Outcomes Quality InitiativeAnemia in chronic kidney disease	1NR	Retrospective single-group cohort	Financial COI disclosed in CPG documents
Hietanen2009 [30]International	NRManagement of early breast cancer	143	Retrospective single-group cohort clinical guidelines in breast cancer	Search for disclosed financial COI via internet and PubMed; The Faculty/Program Committee Disclosure Index of American Society of Clinical Oncology Breast Cancer Symposium 2007; European Society for Medical Oncology
Holloway2008 [13]US	American Academy of NeurologyNeurology, various conditions	50425	Prospective cross-sectional study	Survey of CPG authors
Johnson2009 [25]US	Infectious Diseases Society of AmericaLyme disease, human granulocytic anaplasmosis, and babesiosis	15	Prospective, cross-sectional study	Attorney general anti-trust investigation into the CPG development process
Papanikolaou2001 [28]International	NRVarious clinical conditions	191242	Retrospective single-group cohort	Hand-search of 6 high impact journals for CPGs published at 5-year intervals between 1979 and 1999
Taylor2005 [22]International	NRCPGs with recommendations on prescription medications	215685	Cross-sectional survey	Examined all CPGs involving drugs in the National Guidelines Clearinghouse™ in 2004
**Guidelines on diagnosis**				
Cosgrove2006 [26]US	American Psychiatric Association Manual for the Diagnosis of Psychiatric Disorders (DSM-V)Psychiatry	18170	Retrospective single-group cohort	Review of data from US Patent and Trademark Office, Lexis-Nexis Academic, Medline, other internet search engines; screening between 1989 and 2004 (DSM-IV was published in 1994)
Cosgrove2009 [27]US	American Psychiatric Association Manual for the Diagnosis of Psychiatric Disorders (DSM-V)Psychiatry	NRNR	Retrospective single-group cohort	NR

Abbreviations: COI, conflict of interest; CPG(s), clinical practice guideline(s); DSM, Diagnostic and Statistical Manual for Mental Disorders; NR, not reported; No., number.

Included studies either examined COI at the level of the CPG or at the level of the individual authors of CPGs (Table S2). There were few data on the percentage of CPGs that disclosed information on COI, and the available data suggested that many CPGs do not disclose author COI. Choudhry and colleagues reported that the majority of CPGs (42 of 44) published between 1991 and 1999 did not declare authors' COI [15]. Papanikolaou and colleagues similarly found that only a small percentage (3.7%) of 191 CPGs published in 1999 disclosed COI [28]. In a 2004 review of CPGs in the National Guideline Clearinghouse™ (www.guideline.gov), 42% of CPGs included information on author COI [22]. In the most recent study, 79% of CPGs made no mention of possible competing interests of members [29].

Among CPGs that disclosed COI, the majority involved authors with one or more conflicts. Holloway and colleagues reported that 92% of 50 American Academy of Neurology CPGs had a least one author with a COI and 77% of guideline authors had one or more reported conflicts [13]. *Nature* published results of a 2004 survey of CPGs within the National Guideline Clearinghouse™ that contained pharmacotherapeutic recommendations [22]. Of more than 200 guidelines, only 90 contained information on an individual author's COI, and of those, only 31 were free of industry influence. More than one-third of guideline panels included at least one member who gave seminars on behalf of a relevant drug company. The author of this study expressed concern that guideline authors may underreport COI and reported that the Center for Science in the Public Interest examined the disclosure statements on randomly chosen blood pressure guidelines, and found that several authors did not report relevant sources of research funding [22]. In another study, physicians who produced CPGs had a higher frequency of various payments from industry than physicians who did not develop guidelines (odds ratio 1.41, 95% confidence interval, 1.04 to 1.91) [14].

In a now dated review of CPGs published between 1991 and 1999 on common adult diseases [15], 87% of guideline authors acknowledged some form of interaction with the pharmaceutical industry when questioned on a survey, although most of the CPGs did not report author disclosures (42 of 44 CPGs). On average, authors of treatment guidelines interacted with 10.5 different drug companies and 59% of authors had relationships with companies whose products were specifically considered in those guidelines. Frequent relationships were reported in the three other studies reporting overall percentages of CPG authors with industry relationships: 35% (various clinical topics) [22], 56% (breast cancer management) [30], and 77% (neurology) [13].

All 12 included studies reported financial relationships between guideline authors and the pharmaceutical industry. In addition, a review of the development process of Lyme disease guidelines by the Infectious Diseases Society of America reported relationships between guideline authors and developers of Lyme disease diagnostic test kits and vaccines as well as insurance companies reviewing disability claims related to Lyme disease [25].

Specific types of financial relationships were reported in nine studies (Table S2), the most common being research or salary support and remuneration for consultation or serving on speaker bureaus. The types of financial interests reported by CPG authors were similar across studies. No study examined intellectual COI such as academic advancement, relationships to specialty societies, or previously published study findings or opinions.

We identified no empirical data on the effect of COI on recommendations in CPGs. A survey of perceptions about COI reported that only 7% of CPG authors believed that their own financial relationships with industry influenced their personal conclusions. On the other hand, 19% of CPG authors believed that industry relationships influenced the recommendations of colleagues [15].

One study reported that 42% of CPG authors performed the clinical procedure examined in the CPGs in their practice, with 33% of their clinical effort devoted to such procedures [13]. Several studies noted specific examples whereby the recommendations in the CPG served the disclosed financial interests of the guideline authors (Table S2) [22]–[25], [30].

Two studies examined COI among an expert panel determining diagnostic criteria for disease. Cosgrove and colleagues reported on financial ties among developers of the Diagnostic and Statistical Manual of Mental Disorders (DSM) and reported that 56% (DSM IV [26]) and 68% (DSM V [27]) of panel members had financial interests in the pharmaceutical industry.

## Discussion

There are few studies describing financial COI for CPG authors and the available data suggest that there is a high prevalence of nondisclosure of COI among authors across a variety of clinical specialties, and a high percentage of CPG authors with disclosures report COI. We identified no empirical data on the effect of those conflicts on clinical recommendations within guidelines and no data on intellectual COI or the role of CPG sponsors in guideline development.

Many of the studies in this review examined data that are more than 5 years old, however, and since interest in, and policies relevant to, COI have changed rapidly in recent years, the data examined herein may have limited applicability to CPGs produced in 2011. Since the publication of the studies in this review, marked changes have occurred in awareness of the frequency and potential effects of COI. Physician professional organizations, medical editors, public policy makers, governmental agencies, and industry are all working to address COI among their respective constituents and in their products, including CPGs [31]–[38]. This increased awareness has led to more frequent, transparent, and complete disclosure of financial relationships by authors and sponsors of primary studies and CPGs. Of particular importance is the recent implementation of a uniform, detailed disclosure form by all journals that are members of the International Committee of Medical Journal Editors [31], [39]. A uniform policy for disclosures of competing interests among CPG authors should decrease variation in disclosures, and more importantly, provide both developers and users of CPGs with appropriate information. In addition, the Physician Payment Sunshine provisions, signed into law in March, 2010 as part of the Patient Protection and Affordable Care Act of 2009, require that the U.S. government set up a public database listing any payment or gift to doctors and teaching hospitals valued at more than $10. A publicly available database with this information will be available in 2013 [32]. Furthermore, a recent report by the IOM in the U.S. may provide guidance for developers and publishers of CPGs [40], [41]. Formal processes for translating a body of evidence into recommendations in CPGs (such as the Grading of Recommendations Assessment, Development and Evaluation (GRADE) Working Group) may diminish the effect of COI among guideline panel members on recommendations although data are not yet available to affirm this [42]. Thus in 2011, there is certainly increasing attention paid to COI, but data are lacking on the current prevalence of COI in recently developed CPGs and on the effects of COI on recommendations.

We noted significant variation in the prevalence of COI among CPG authors, which might be explained by different policies across journals and organizations sponsoring CPGs, by variations in culture and funding of CPGs among specialties, and by the different time periods for development of guidelines examined in studies included in this review.

There are a large number of gaps in the available literature. We were unable to identify any studies that examined the actual effect (not just the prevalence) of industry affiliations or support on recommendations in CPGs. This is perhaps not surprising as it is very difficult to qualify, let alone quantify, the effects in observational studies of industry relationships on conclusions because confounders as well as of other sources of bias in addition to COI. In addition, observational data on COI cannot, of course, prove that conflicts are causally related to specific recommendations. Nonetheless, observational data can be used to explore relationships and to generate hypotheses on the extent and direction of the influences exerted by specific conflicts on recommendations in CPGs. The frequent lack of temporal data within financial disclosures also makes it difficult to explore associations between COI and CPG recommendations.

We identified no data on the nature, extent, and effect of intellectual COI. This type of COI is much more difficult to define and quantify, let alone examine its effect on specific recommendations. It has been suggested, however, that intellectual COI may be a far more important influence on guideline developer decision making than financial interests [19], [43]. Few CPG organizations are reporting this type of COI, although there is some interest in developing policies addressing this issue [18].

In addition, we found no evidence on the role of sponsors or funders in CPG development and decision making. The IOM Panel reported that medical specialty societies (40.9%) and professional associations (17.4%) are the dominant funders of CPGs contained within the National Guideline Clearinghouse™ [44]. These institutions could potentially be a major source of bias in the generation of CPG recommendations if they do not have adequate and transparent quality controls and processes in place. The sponsor may be in a position to influence the selection of CPG topics, the identification and evaluation of the body of evidence, the process for deriving recommendations from the evidence, and dissemination of the CPGs [44]. The influence of industry sponsorship or author interests may be particularly important when data for CPG recommendations are lacking, as is frequently the case [45].

### Limitations

There was heterogeneity of methodologies used for the assessment of COI, thus we were unable to perform a quantitative synthesis (meta-analysis) of the data. Some studies examined disclosures reported by CPG authors, while other studies reported author financial interests gleaned from internet searches. Classification of COI and methods for reporting data also differed across studies.

A further limitation of the available data was that the accuracy of disclosures, both completeness and specific relationships reported, was not assessed in the studies reviewed herein. Self report by guideline authors may be inaccurate, as suggested by data on disclosures in primary research studies [46] and responses rates were low when surveying CPG authors on their COI [15]. Some of the studies in this review explored financial relationships for CPG authors in addition to information provided in author disclosures [24], [26], [30], but comparisons were not made between information from these two sources. Internet searches for COI may also be incomplete or inaccurate as they will likely not reveal all important funders and sponsors, and certainly will not reveal stock and other equity relationships.

In addition to the limitations imposed by the available data, our study had limitations in methodology. First, we searched Medline for studies for inclusion, and it is possible that other bibliographic databases might have provided additional studies. We felt, however, that other databases would likely have very low incremental yield given that we focused on English-language CPGs, performed an extensive MeSH and text work search, and reviewed reference lists of included studies and background papers. We searched for non-English studies indexed in Medline and found none, suggesting that most of the relevant literature is in the English language and thus more likely to be identified in Medline. Second, we did not perform dual independent review of data abstraction; rather a second author checked all abstractions.

### Future Research

Much additional research is needed on the nature and impact of COI in the development of CPGs. Research is needed on the effect of CPG authors' financial and intellectual interests on their decision processes, their assessment of the quality and inclusion of specific studies in the body of evidence, their assessment of the direction and strength of the evidence, and the translation of evidence into recommendations. The accuracy of disclosures of financial interests by CPG authors needs to be examined. Further work is needed as to whether formal processes such as GRADE reduce bias in guideline development associated with COI among the CPG authors [42]. Work is also needed on how disclosed COI affect readers' perceptions. Research is needed on the potential effects of intellectual COI. The role of sponsors in the selection of guideline panel members and in the processes and generation of recommendations needs to be explored, along with the risk for bias in CPG recommendations due to specialty and other professional and personal interests. Little is known about how biomedical journal readers interpret disclosures and whether and/or how they actually apply the information [47]–[50]. Such empirical data will then be available to guide future policies on optimal processes for collection of accurate disclosures and the subsequent management of disclosed conflicts, as well as methods for presenting meaningful information to users of CPGs.

CPGs are designed to be widely used, to impact healthcare provider practice, to have positive effects on economic efficiency, and to ultimately improve patient outcomes. Until such time as CPGs uniformly report accurate and relevant disclosures, there are data available on the relationships between various types of COI and guideline recommendations, and such relationships are appropriately managed, the user of CPGs is at a severe disadvantage in evaluating the quality of a given CPG. For now, users need to critically appraise CPGs considered for implementation, read disclosures and consider how they may have influenced recommendations, and seek to move forward research on unanswered questions.

## Supporting Information

Figure S1 PRISMA flow diagram 1980 to March, Week 4, 2011.
(DOCX)

Table S1 Search strategy. Ovid MEDLINE and Ovid OLDMEDLINE 1980 to March, Week 4, 2011.
(DOCX)

Table S2 Results of included studies. Abbreviations: CI, confidence interval; COI, conflict of interest; CPG(s), clinical practice guideline(s); ESP, erythropoietin-stimulating protein; NR, not reported; No., number; OR, odds ratio.
(DOCX)
