# Supplementary material for: Conflict of Interest in Clinical Practice Guideline Development: A Systematic Review
Source: PLoS One. 2011 Oct 19;6(10):e25153. doi: 10.1371/journal.pone.0025153 (PMC3198464; doi:10.1371/journal.pone.0025153)
Supplement: Figure S1 — PRISMA flow diagram 1980 to March, Week 4, 2011. (DOCX) [file pone.0025153.s001.docx]

Figure S1. PRISMA Flow Diagram 1980 to March, Week 4, 2011

Studies included in qualitative synthesis
(n=12 )

Full-text articles excluded
(n=196 )

Full-text articles assessed for eligibility
(n=208 )

Records excluded
(n=791)

Records screened
(n=999)

Records after duplicates removed
(n=999)

Additional records identified through other sources
(n=10)

Records identified through database searches
(n=989)
