# Supplementary material for: Conflict of Interest in Clinical Practice Guideline Development: A Systematic Review
Source: PLoS One. 2011 Oct 19;6(10):e25153. doi: 10.1371/journal.pone.0025153 (PMC3198464; doi:10.1371/journal.pone.0025153)
Supplement: Table S1 — Search strategy. Ovid MEDLINE and Ovid OLDMEDLINE 1980 to March, Week 4, 2011. (DOCX) [file pone.0025153.s002.docx]

Table S1. Search Strategy

Ovid MEDLINE(R) and Ovid OLDMEDLINE(R) 1980 to March Week 4, 2011

| **#** | **Searches** |  |
| --- | --- | --- |
| 1 | exp "Conflict of Interest"/ |  |
| 2 | (conflict adj3 interest$).mp. [mp=title, original title, abstract, name of substance word, subject heading word, unique identifier] |  |
| 3 | Drug Industry/es, lj [Ethics, Legislation & Jurisprudence] |  |
| 4 | Research Support as Topic/es, lj, st [Ethics, Legislation & Jurisprudence, Standards] |  |
| 5 | exp guidelines as topic/ |  |
| 6 | (practice adj3 guideline$).mp. [mp=title, original title, abstract, name of substance word, subject heading word, unique identifier] |  |
| 7 | (clinical adj guideline$).mp. [mp=title, original title, abstract, name of substance word, subject heading word, unique identifier] |  |
| 8 | or/1-4 |  |
| 9 | or/5-7 |  |
| 10 | 8 and 9 |  |
| 11 | limit 10 to (humans and yr="1980 -Current") |  |
