# Supplementary material for: Conflict of Interest in Clinical Practice Guideline Development: A Systematic Review
Source: PLoS One. 2011 Oct 19;6(10):e25153. doi: 10.1371/journal.pone.0025153 (PMC3198464; doi:10.1371/journal.pone.0025153)
Supplement: Table S2 — Results of included studies. Abbreviations: CI, confidence interval; COI, conflict of interest; CPG(s), clinical practice guideline(s); ESP, erythropoietin-stimulating protein; NR, not reported; No., number; OR, odds ratio. (DOCX) [file pone.0025153.s003.docx]

**Table S2**: Results of included studies

| **Author**  **Year** | **Prevalence of COI among CPGs** | **Prevalence of COI among CPG authors** | **Types of industry relationships** | **Effect of COI**  **Predictors of Disclosures** |
| --- | --- | --- | --- | --- |
| **Guidelines on treatment and prevention** | | | | |
| Buchan  2010 [29] | CPGs that did not mention competing interests of the CPG development group (n=313): 79% | NR | NR | Competing interest policies on websites of the organizations that produced or co-produced ≥ 5 CPGs (n=22) (% of organizations):  policies specific to CPG development: 18%; general competing interest policies applying to committees, authorship or relationships with pharmaceutical industry: 36%; no information about competing interest policies: 45%. |
| Campbell  2007 [14] | NR | NR | CPG developers versus physicians not involved in CPGs (OR (95% CI)): Samples: 0.74 (0.53-1.03) Gifts: 0.92 (0.64-1.33) Reimbursements: 1.08 (0.83-1.42) Payments: 1.41 (1.04-1.91) | NR |
| Choudhry  2002 [15] | No COI declarations (n=44): 95% | CPG authors with any relationship with the pharmaceutical industry: 87%  Mean number of companies with which authors interacted: 10.5 (range, 1-37) | % authors (95% CI) / mean number of companies (range)   Travel funding/honorarium: 53 (43-63) / 5.4 (1-16) Speaker honorarium: 64 (54-74) / 7.3 (1-20) Educational program support: 51 (41-61) / 4.7 (1-36) Research support: 58 (48-68) / 6.7 (1-26) Employee/consultant: 38 (28-48) / 5.7 (1-21) Equity: 6 (1-11) / 1.8 (1-4) | Authors with relationships with companies whose products were specifically considered in the CPG: 59%  Authors who believed that relationships influenced personal recommendations: 5/68 (7%) (95% CI, 1-9)  Authors who believed that relationships influenced recommendations of colleagues: 13/67 (19%, 95% CI, 8-30) |
| Cosgrove  2009 [24] | NR | One or more financial ties to pharmaceutical industry by CPG authors: 90% (none were disclosed in the CPGs)  Work group members for schizophrenia and bipolar CPGs with industry ties: 100% | Types of financial relationships (n=18 authors) Research funding: 77.7%  Consultancy: 72.2%  On corporate or company advisory boards: 44.4% Honoraria: 38.8%  Served on company speaker's bureaus: 33.3% Equity in a relevant company: 16.6%   Number of categories of financial interest in the pharmaceutical industry for each author:  >1: 88.8%  ≥ 3: 66.6%  ≥ 4: 55.5% | In 100% of authors who had industry relationships, these relationships were with companies whose products were specifically considered or included in the guideline they authored.  100% of companies whose drugs were listed as 'optimal' for manic depressive disorder provided funding to CPG authors.   100% of authors of CPGs on schizophrenia had financial relationships with companies whose drugs were identified as 'commonly used'. |
| Coyne  2007 [23] | NR | Workgroup members who reported receiving consultant fees, speaking fees, and/or research funds from at least one company that produces ESP drugs: 87.5%  Members who reported potential COI with the ESP industry: 68.8% | NR | The recommendation for an increase in target hemoglobin will increase ESP drug use although benefits versus risks were unclear and two unpublished trials were excluded from consideration. |
| Hietanen  2009 [30] | NR | Expert panel members with relationship to companies that market aromatase inhibitors (n=43): Any potential COI: 55.8%  No potential COI or no relevant articles: 44.2% | Expert panel members with relationship to companies that market aromatase inhibitors (n=43):  Stock ownership: 2.3% Consultant or advisory role: 41.9% Honoraria/lecture fees/other remuneration: 41.9% Research funding: 39.5% | Aromatase was voted to be used as the initial treatment in early breast cancer. There is no evidence of superiority of aromatase over the cheaper tamoxifen for initial treatment of women with hormone-responsive breast cancer. In addition, tamoxifen has a well known safety profile. |
| Holloway  2008 [13] | CPGs with ≥ 1 author with a COI: 92% | CPG authors with any COI: 77%  Authors with multiple COI:  3-5: 28%; 6-10: 15%; >10: 5% | Types of COI among CPG authors (n=351)  Research: 45%  Performed a relevant procedure in clinical practice: 42% Clinical effort related to a CPG procedure: 33% Equity/stock options: 7% Expert testimony: 6% Fiduciary role: 3% Advocacy role: 2% Patent rights/royalties: 2% | NR |
| Johnson  2009 [25] | NR | COI disclosed by key CPG panel members (n=5): 100% | Lyme diagnostic tests: 60%  Lyme vaccines: 100%  Insurance consulting/expert testimony: 80% | CPG recommendations were favorable to vaccine manufacturers, test kit developers, and insurance companies. |
| Papanikolaou  2001 [28] | CPGs disclosing COI (all disclosures were in 1999): 3.7% | Authors disclosing a COI in 1999: 17.5% | Type of COI among authors with disclosures (n=18): Paid travel fees: 29% Consultation: 21% Received grants: 13% Speaker fees: 13% Attended symposia: 8% Salary support: 4%  Worked for a specific company: 8% Contract for a research project: 4% | CPGs funded mostly by the government, universities, or major professional organizations almost never reported potential COI while CPGs funded by private or mixed sources were more likely to disclose COI (p<0.001)  Increasing no. of authors correlated with no. of disclosed COI (P=0.004); reporting was related to specific journals (p=0.026) |
| Taylor  2005 [22] | CPGs reporting on author's COI (n=215 CPGs): 42%  Of CPGs with disclosures (n=90), % that declared no COI for any author: 34% | Among authors who provided disclosures (n=685), declared no COI: 65% | Of CPGs with author disclosures (n=90 CPGs):  ≥ 1 author had an advisory board or consultancy with company: 50%  ≥ 1 author received research funding from company: 50%  ≥ 1 author had a speaker's bureau position: 43%  ≥ 1 author owned stock in company: 11%  Nature of disclosed COI by authors (n=685):  Research grant:21%  Advisory board or consultancy role: 22%  Speaker's bureau: 15%  Owned stocks: 2% | NR |
| **Guidelines on diagnosis** | | | | |
| Cosgrove  2006 [26] | NR | One or more of 11 categories of financial interests in a pharmaceutical company (n=170 authors): 56%  >80% of members had financial ties to industry in 1/3 of diagnostic panels  Multiple financial interests:  Authors disclosing in 2 categories: 16%; 5 categories: 12% | Among 170 panel members: 42% had drug industry research funding, 22% were consultants, 16% were on drug company's speaker bureau. | NR |
| Cosgrove  2009 [27] | NR | Members of task force with industry ties: 68%  Panel members with posted disclosure statements (n=137) who reported industry ties: 56% | NR | NR |
